# Effectiveness of community-based culturally tailored cervical cancer awareness interventions among women and decision-making men in low-resource settings: A pre-post design evaluation in rural Uganda and Bangladesh

**DOI:** 10.1016/j.pmedr.2026.103575

**Published:** 2026-07-15

**Authors:** Fareezah Abdul Karim Siddique, Janine de Zeeuw, Jaap A.R. Koot, Carolyn Nakisige, Naheed Nazrul, Dan Atukonyera, Geertruida H. de Bock, PRESCRIP-TEC consortium

**Affiliations:** a Department of Epidemiology, University Medical Center Groningen, University of Groningen, the Netherlands; b Global Health Unit, Department of Health Sciences, University Medical Center Groningen, University of Groningen, the Netherlands; c Department of Gynaecologic-Oncology, Uganda Cancer Institute, Kampala, Uganda; d Health Sector, FRIENDSHIP NGO, Dhaka, Bangladesh; e Uganda Rural Development Training Program, Kagadi, Uganda; f Health System and Population Studies Division, ICDDR, B, Dhaka, Bangladesh; g Department of Gynaecology, Leiden University Medical Centre, Leiden, The Netherlands; h Usher Institute, University of Edinburgh, Edinburgh, Scotland, United Kingdom EH16 4UX

**Keywords:** Uterine cervical neoplasms, Early detection of cancer, Health education, Low resource setting, Developing countries, Rural population, Mass media

## Abstract

**Objective:**

Cervical cancer disproportionately affects women in low-resource settings (LRS), where screening uptake and awareness remains low. Male household decision-makers often influence women's health decisions but are infrequently targeted in awareness interventions. This study evaluated culturally tailored interventions to improve cervical cancer awareness among women and male household decision-makers in rural Uganda and Bangladesh.

**Methods:**

A quasi-experimental pre-post design using data from the PRevention and SCReening Innovation Project Toward Elimination of Cervical Cancer (PRESCRIP-TEC) surveyed women and male household decision-makers before (Uganda May 2022; Bangladesh April–June 2022) and after (December 2022–October 2023) a mass media with community health worker (CHW) outreach intervention. Multivariable logistic regression assessed changes in risk factor and symptom knowledge.

**Results:**

Among 3342 respondents (1453 pre-, 1889 post-intervention), women and decision-making men showed significantly improved awareness post-intervention: risk factor knowledge (women OR = 9.51, 95% CI: 7.52, 12.02; men OR = 11.72, 95% CI: 8.03, 17.10) and symptom knowledge (women OR = 6.24, 95% CI: 5.03, 7.74; men OR = 4.53, 95% CI: 3.31, 6.19), after adjustment for covariates.

**Conclusions:**

Culturally tailored interventions engaging male household decision-makers alongside women and integrating mass media with CHW-led outreach effectively improves cervical cancer awareness across socio-culturally diverse LRS and can inform policy and screening campaigns in LRS.

## Highlights

• Cervical cancer awareness improved in both women and men in low-resource settings.
• Culturally tailored outreach and media improved awareness in diverse settings.
• Among few studies to assess male awareness change in low-resource settings.
• Findings support scaling culturally adapted interventions in low-resource settings.

## 1 Introduction

Cervical cancer is the fourth most common cancer affecting women worldwide, with approximately 660,000 cases and 350,000 deaths in 2022 (Bray et al., 2024). Over 80% of both cases and deaths occur in low- and middle-income countries (LMICs), highlighting a significant global disparity (Ferlay et al., 2024). Cervical cancer is largely preventable given its primary cause, persistent infection of high-risk human papillomavirus (hrHPV) (Burd, 2003). Timely screening (e.g., hrHPV self-sampling) followed by early treatment, significantly improves survival (Peirson et al., 2013). The World Health Organization's (WHO) 2021 cervical cancer elimination strategy set a 2030 goal of 70% screening and 90% treatment (World Health Organization, 2021). Yet despite these ambitions, two-thirds of women worldwide aged 30–49 have never been screened and are frequently diagnosed at advanced stages of cervical cancer (Bruni et al., 2022). Within LMICs, rural women are screened less often and have higher cervical cancer rates than urban women, pointing to growing intra-country disparities (Garg et al., 2025; Lemp et al., 2020). These inequalities reflect deeper challenges limiting access and uptake of screening, especially in rural remote low-resource settings (LRS) within LMICs.

Awareness of cervical cancer risk factors and symptoms is a key barrier to screening participation (Chinn and Tewari, 2020), often shaped by broader challenges of sociocultural norms, low education, low socioeconomic resources, and inadequate and inaccessible healthcare resources (Petersen et al., 2022; Zhang et al., 2022). Misconceptions and cultural taboos about the disease distort understanding of its risks (Maseko et al., 2024). This is especially true for men, who are often less aware of cervical cancer than women, yet as husbands or partners, they often serve as primary decision-makers to women's screening (Ngwenya and Huang, 2018; Rawat et al., 2023). However, most cervical cancer awareness interventions in LRS continue to solely target women. Some interventions in LRS which involve men, include community health workers (CHWs) using home visits or location-based sessions (e.g., study sites or locations near clinics) (Basu et al., 2019; Budukh et al., 2024; Chinula et al., 2021; Lidofsky et al., 2019; Vahabi et al., 2023). These approaches, while effective, may miss hard-to-reach groups, and can limit effectiveness without broader community engagement. Combining CHW-led outreach with community-tailored mass media approaches can broaden reach, reinforce key messages, help address community-wide stigma, misconceptions and low awareness more comprehensively (Bawuah et al., 2025). To our knowledge, no prior study in LRS has evaluated a combined mass media and CHW-led awareness intervention for hrHPV self-sampling that measures post-intervention awareness beyond women alone to include male household decision-makers.

Addressing this gap is critical in LRS where opportunistic screening policies mean that community-based awareness often represents the main route through which women and their households obtain information about cervical cancer screening. Rural areas of Uganda and Bangladesh were selected to illustrate that, despite substantial geographical and cultural differences, both settings face similar barriers to screening participation that are common across LRS. Thus, the objective of this study is to evaluate an intervention combining mass media campaigns with CHW-led outreach methods, among both women and male household decision-makers and tailored to the contexts of two LRS to improve cervical cancer awareness. By assessing the effectiveness of this context-tailored, combined approach across diverse LRS, the study provides insight into transferable strategies to improve awareness more broadly. We hypothesised that such an intervention will significantly increase cervical cancer awareness post-intervention in females and male household decision-makers living in LRS.

## 2 Methods

### 2.1 Study design and population

This study was conducted within the PRevention and SCReening Innovation Project Toward Elimination of Cervical Cancer (PRESCRIP-TEC) (Sultanov et al., 2022; Nazrul et al., 2024; Nakisige et al., 2024). PRESCRIP-TEC aimed to evaluate the feasibility of 2021 WHO guidelines by implementing primary hrHPV self-sampling in LRS, which includes rural Bangladesh and rural Uganda. PRESCRIP-TEC implementation activities included:

1. community sensitisation activities through mass media campaigns and CHW-led outreach among women and household decision-makers, and tailored to local contexts to raise awareness about cervical cancer screening and treatment,
2. hrHPV self-sampling offered at home by CHW and,
3. building local health system capacity for visual inspection with acetic acid triage (Bangladesh) or for treatment assessment (Uganda).

The following catchment areas were included per country as these were selected as part of the PRESCRIP-TEC project (Nazrul et al., 2024; Nakisige et al., 2024):

• Uganda (mid-Western region): Kakumiro with sub-districts including Mpassaana, Katikara, Kitaihuka, Birembo and Kisengwe.

Bangladesh (northern and southern region): Gaibandha and Kurigram in the north, and Sathkira intervention areas in the south. For the presented analysis, the focus is on the awareness activities using a quasi-experimental design with different subpopulations surveyed pre- and post-intervention. These groups were considered comparable, as participants were recruited from the same rural areas using identical eligibility criteria.

**Pre-intervention survey.** Prior to the PRESCRIP-TEC implementation, pre-intervention surveys were conducted in eligible catchment areas in each country. Findings, including the prominent role of male household decision-makers in women's health decisions, directly informed intervention development, with detailed results reported in country-specific publications (Nazrul et al., 2024; Nakisige et al., 2024). This survey lasted around 1–3 months:

• Uganda: 2nd – 20th May 2022;
• Bangladesh: 24th April – 19th June 2022.

**Intervention.** The intervention comprised two components: mass media campaigns and CHW-led outreach. The tailoring and activities per component are described below, with further details of each activity in the supplementary materials. The intervention was implemented using a phased geographic rollout across catchment areas, with evaluation surveys scheduled relative to local implementation timing rather than after completion across the entire study region.

***Cultural tailoring:*** Activities and materials for both components were culturally-tailored by adapting communication channels, community engagement strategies, and messages to local sociocultural, literacy, and resource conditions, informed by pre-intervention survey findings and local implementing partners' input. PRESCRIP-TEC consortium partners identified literacy levels and socio-cultural barriers (e.g., cervical cancer stigma, misconceptions, and reported household involvement in women's health decisions) to cervical cancer screening uptake. Various media formats were tested for reach and most engagement under local resource constraints (e.g., limited internet and/or electricity). Strategies leveraged existing networks and employed trained CHWs from target communities. Local teams received in-house training from PRESCRIP-TEC partnered organisations and a communication partner specialising in event organization and social media marketing. The sensitisation messages delivered covered cervical cancer symptoms, risk factors, and screening information, corresponding to the awareness outcomes evaluated.

***Mass media campaigns:*** For both settings, this included posters in health and community centres, leaflets, roll-ups, banners, news articles, advice and information through PRESCRIP-TEC website, social media posts, and YouTube short videos. Uganda additionally implemented weekly local radio talk shows and newspaper advertisements in the Kakumiro district.

***CHW-led outreach:*** For both settings included household door-to-door visits and community awareness sessions. For Bangladesh, open community village theatrical performances, and sessions with community opinion leaders (e.g., religious leaders and influential figures). These leaders organised neighbourhood meetings with household decision-makers. During household visits, CHWs provided cervical cancer information to eligible women and identified male household decision-makers (husbands or family members). Hr-HPV self-sampling was offered by CHWs using a door-to-door strategy in both countries (PRESCRIP-TEC, 2024).

The country-specific intervention dates for awareness raising and hrHPV swab collection were as follows:

• Uganda: 31st May 2022 – 5th October 2023
• Bangladesh: 12th June 2022 – 4th March 2023

**Post-intervention survey:** Participants were re-sampled from the same intervention areas using identical eligibility criteria, with survey data collected 6–12 months after pre-intervention surveys. Country-specific intervention dates are as follows:

• Uganda: 10th May 2023 – 29th October 2023
• Bangladesh: 12th December 2022 – 13th April 2023

Post-intervention dates were conducted after implementation within each area; therefore, some survey dates precede the overall intervention end date for the country.

**Population:** Female participants were eligible if they met pre-defined age groups in accordance with PRESCRIP-TEC cervical cancer screening practices, as determined by local implementing partners: Bangladesh (30–60 years) and Uganda (30–49 years). Exclusion criteria included pregnancy, a history of hysterectomy, current signs, and symptoms suggestive of cervical cancer, prior ablative or excisional treatment for cervical cancer, or vaginal infection. Eligible females and male decision-makers from the same household had to provide written or oral informed consent with a representative signing for oral consent. Male household decision-makers were identified during recruitment by asking the enrolled woman to indicate the adult male household member (e.g., husband or senior male family member) involved in decisions regarding her health, who was then invited to participate. They were also required to understand and respond to survey questions, and willing to be interviewed privately. Country-specific sampling procedures are detailed in pre-intervention publications (Nazrul et al., 2024; Nakisige et al., 2024), and the same methods were applied during the post-intervention phase. All participants who completed the AWACAN at pre- or post-levels were included in the presented analysis in this study.

### 2.2 Measures

Surveys included demographic and awareness questionnaires at both pre- and post-intervention levels. Surveys are provided in the supplementary materials.

**Demographics**: These included sex, age, education status, and marital status. Education was grouped as “No education” (no formal education or incomplete primary school) and “Primary education and higher.” Marital status was collapsed into “with” (married or cohabiting) or “without” partner (never married/single, divorced, separated, or widowed). Three female-specific demographic questions were also included: 1. The lifetime CC screening history (a participant would respond “yes/no” on whether a healthcare worker ever screened them for cervical cancer). 2. Women were asked about who made decisions regarding their health - they could select the following: “myself”, “partner only”, “myself and partner”, “someone else in the household”, or “myself and someone else in the household”. This was collapsed into “myself” or “not myself/joint decision-making” to harmonise differing country options and avoid sparse categories in pooled analyses. 3. In the post-intervention survey, women were asked whether they participated in the hrHPV self-sampling (“yes/no”).

**Awareness and primary outcome:** Cervical cancer awareness was measured using the adapted cervical cancer sub-section of the African Woman Awareness of CANcer (AWACAN) (Moodley et al., 2019). This is the primary outcome of the presented analysis. This 52-item validated questionnaire assesses distinct domains including risk factors, symptoms, lay beliefs, appraisal confidence, help-seeking behaviours, and barriers to accessing health care. The analysis focused on risk factor and symptom knowledge domains as measures of awareness, consistent with prior applications of their use (Govender et al., 2025; Adoch et al., 2020). Other domains capturing beliefs and behaviours were beyond the scope of this evaluation. The measure demonstrated good internal reliability measured using the Kuder-Richardson formula for symptom knowledge (0.80) and moderate reliability for risk factor knowledge (0.60) (Moodley et al., 2019). Participants responded “Yes” (coded as 1), “No”, or “I don't know” (both coded as 0) to items on cervical cancer risk factors (15 items) and symptoms (12 items). AWACAN scores reflect correct recognition of established risk factors and symptoms; therefore, although more items are presented, domain scores range from 0 to 11. Risk factor items were asked only among participants reporting prior awareness of cervical cancer. Higher numbers indicate better awareness of cervical cancer risk factors and symptoms, respectively. Questionnaires were contextualised and translated using forward and backward translation. The questionnaire is provided in DataverseNL (Abdul Karim Siddique et al., 2025). The AWACAN and its underpinning measure (Stubbings et al., 2009) were developed on the premise that awareness of risk factors and symptoms, while not sufficient alone, is a necessary precondition for behaviour change. This is consistent with cognitive-behavioural theories of health promotion (National Cancer Institute, 2005) whereby knowledge is the foundational step toward informed decision-making and screening participation.

### 2.3 Statistical analysis

Baseline characteristics and awareness scores were described at pre−/post-levels and separately for female and males. Due to skewness, continuous variables were dichotomized at the median. This included awareness scores, which showed a pronounced floor-effect with many zeros. Thus, binary logistic regression was used to distinguish lower versus higher awareness while allowing adjustment for relevant covariates. Prior to performing the logistic regression analyses, we analysed missing data patterns and applied multiple imputations (*n* = 5) to fill in for missing values. Multicollinearity was assessed and all values were within the acceptable thresholds. Thereafter, univariate, and multivariable analyses were performed for both sexes, where pre−/post-intervention level, country, age, education, marital status were used as predictors to risk factor and symptom knowledge scores. Lifetime cervical cancer screening and health decision-making status were an added predictor in the female-only models. The selection of covariates (i.e., country, age, education level, and marital status) were informed by a combination of univariate analyses and literature. Finally, a complete case analysis was conducted as a sensitivity analysis. All analyses used an alpha of 5% for significance and were conducted using Statistical Package for Social Sciences® version 26. Data visualization was conducted using R version 4.5.1.

**Ethical Compliance**: The study was registered in ClinicalTrials.gov, NCT05234112. Favourable Ethical Opinion for PRESCRIP-TEC activities were obtained from the country-specific research ethics committees:

• Bangladesh: Institutional Review Board of International Centre for Diarrheal Disease Research, Bangladesh (ICDDR, B), approval number PR-21029, approved on 30th January 2022;
• Uganda: Uganda Cancer Institute Research Ethics Committee, registration number: UCI-2021-29, approved on 5th April 2022.

This study was conducted in accordance with institutional guidelines for the protection of human subjects concerning safety and privacy.

## 3 Results

### 3.1 Population characteristics

Table 1 shows the population characteristics. The sample consisted of 1453 pre-intervention respondents and 1889 post-intervention respondents (57%). In total, 2199 females (66%) and 1143 male household decision-makers from both LRS across both pre−/post-intervention levels were included. Females were aged between 30 and 60 years (*M*_Age_ = 37.11 years, *SD*_Age_ = 6.09), while male household decision-makers were aged between 20 and 79 years (*M*_Age_ = 41.36 years, *SD*_Age_ = 7.95). Respondents often reported having a partner and having no education. Females in both countries often reported that they were not autonomous decision-makers regarding their health. Overall, the characteristics between the pre- and post-intervention samples were similar, although there were differences in education level for both sexes. Awareness indicators were higher at post-intervention, with some variation between countries. Cervical cancer screening prevalence was lowest at pre-intervention level and increased following the intervention and hrHPV self-sampling.

**Table 1:** Demographic characteristics and cervical cancer awareness indicators stratified by country and pre- and post-intervention levels among women and male household decision-makers across low resource settings in Uganda and Bangladesh (2022−2023).

			Rural Uganda		Rural Bangladesh		Total
Females			Pre- (*N* = 423)	Post- (*N* = 967)	Pre- (*N* = 298)	Post- (*N* = 511)	Females (*N* = 2199)
Age^a^ n, %							
	< 36 years		147, 34.8%	546, 56.5%	137, 46.0%	183, 35.8%	1013, 46.1%
	≥ 36 years		208, 49.2%	421, 43.5%	161, 54.0%	328, 64.2%	1118, 50.8%
	Missing		68, 16.1%	..	..	..	68, 3.1%
Marital status n, %							
	No partner		51, 12.1%	41, 4.2%	..	10, 2.0%	102, 4.6%
	Partner		305, 72.1%	926, 95.8%	298, 100%	501, 98.0%	2030, 92.3%
	Missing		67, 15.8%	..	..	..	67, 3.0%
Highest level of education n, %							
	No education		254, 60.0%	624, 64.5%	275, 92.3%	275, 53.8%	1428, 64.9%
	Primary Education and Higher		102, 24.1%	343, 35.5%	23, 7.7%	236, 46.2%	704, 32.0%
	Missing		67, 15.8%	..	..	..	67, 3.0%
Health Decision-maker n, %							
	Myself		114, 27.0%	133, 13.8%	8, 2.7%	13, 2.5%	268, 12.2%
	Not myself/Joint decision-making		309, 73.0%	832, 86.0%	289, 97.0%	498, 97.5%	1928, 87.7%
	Missing		..	2, 0.2%	1, 0.3%	..	3, 0.1%
Risk Factor knowledge^a^ n, %							
	Low scores		289, 68.3%	370, 38.3%	260, 87.2%	151, 29.5%	1070, 48.7%
	High scores		112, 26.5%	560, 57.9%	38, 12.8%	360, 70.5%	1070, 48.7%
	Missing		22, 5.2%	37, 3.8%	..	..	59, 2.7%
Symptom knowledge^a^ n, %							
	Low scores		255, 60.3%	199, 20.6%	198, 66.4%	138, 27.0%	790, 35.9%
	High scores		135, 31.9%	741, 76.6%	100, 33.6%	373, 73.0%	1349, 61.3%
	Missing		33, 7.8%	27, 2.8%	..	..	60, 2.7%
Lifetime cervical cancer screening prevalence n, %			28, 6.6%	394, 40.7%	29, 9.7%	66, 12.9%	517, 23.5%
	Missing		16, 3.8%	27, 2.8%	1, 0.3%	11, 2.2%	55, 2.5%
Uptake of high-risk human papillomavirus self-sampling^b^ n, %			..	618, 63.9%	..	467, 91.4%	1085, 73.4%
	Missing		..	317, 32.8%	..	25, 4.9%	342, 23.1%

Male household decision-maker			Pre- (*N* = 432)	Post- (*N* = 257)	Pre- (*N* = 300)	Post- (*N* = 154)	Males (*N* = 1143)
Age^a^ n, %							
	< 40 years		237, 54.9%	100, 38.9%	94, 31.3%	26, 16.9%	457, 40.0%
	≥ 40 years		149, 34.5%	157, 61.1%	206, 68.7%	126, 81.8%	638, 55.8%
	Missing		46, 10.6%	..	..	2, 1.3%	48, 4.2%
Marital status n, %							
	No partner		71, 16.4%	2, 0.8%	..	..	73, 6.4%
	Partner		313, 72.5%	255, 99.2%	300, 100%	152, 98.7%	1020, 89.2%
	Missing		48, 11.1%	..	..	2, 1.3%	50, 4.4%
Highest level of education n, %							
	No education		285, 66.0%	82, 31.9%	259, 86.3%	92, 59.7%	718, 62.8%
	Primary Education and Higher		102, 23.6%	175, 68.1%	41, 13.7%	60, 39.0%	378, 33.1%
	Missing		45, 10.4%	..	..	2, 1.3%	47, 4.1%
Risk Factor knowledge^a^ n, %							
	Low scores		222, 51.4%	17, 6.6%	201, 67.0%	27, 17.5%	467, 40.9%
	High scores		176, 40.7%	220, 85.6%	95, 31.7%	125, 81.2%	616, 53.9%
	Missing		34, 7.9%	20, 7.8%	4, 1.3%	2, 1.3%	60, 5.2%
Symptom knowledge^a^ n, %							
	Low scores		208, 48.1%	44, 17.1%	173, 57.7%	45, 29.2%	470, 41.1%
	High scores		199, 46.1%	207, 80.5%	120, 40.0%	107, 69.5%	633, 55.4%
	Missing		25, 5.8%	6, 2.3%	7, 2.3%	2, 1.3%	40, 3.5%

**Note.**^a^Age, risk factor knowledge and symptom knowledge were split at the median. ^b^High-risk human papillomavirus self-sampling was offered during the intervention and post-intervention period. Pre- and post-intervention surveys sampled different participants, changes in distributions should not be interpreted as within-household change.

### 3.2 Cervical cancer awareness improvement in LRS

**Fig. 1:**
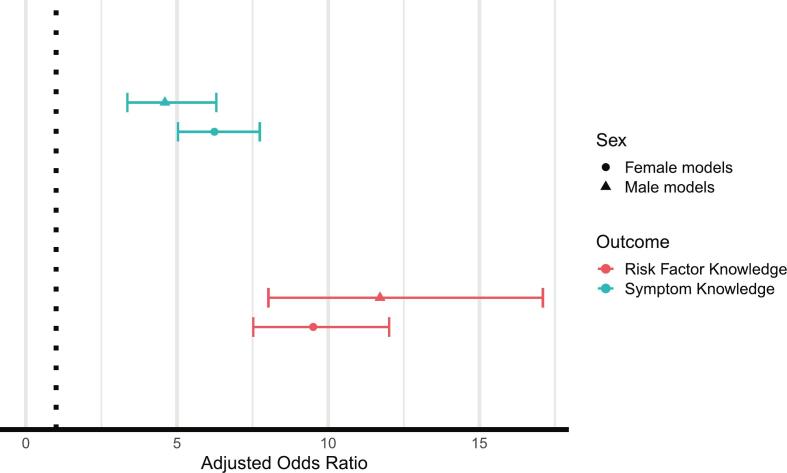
Forest plot of adjusted odds ratio and 95% confidence intervals for the association between pre- and post-intervention status and cervical cancer awareness outcomes among women and male household decision-makers across low resource settings in Uganda and Bangladesh (2022–2023). ***Note.*** Pre-intervention is used as the reference group. All models adjust for country, age, education, and marital status. Female models additionally adjust for health decision-making status and lifetime cervical cancer screening.

Univariate analyses for both females and male household decision-makers are provided in the supplementary table S1. Fig. 1 displays the multivariable association between post-intervention status and cervical cancer awareness outcomes, by sex. Compared to the pre-intervention group, post-intervention women had significantly higher odds of scoring high on both risk factor (*N* = 2140, OR = 9.51) and symptom knowledge (*N* = 2139, OR = 6.24), after adjusting for country, age, education, marital status, women's health decision-making status, and prior cervical cancer screening.

Among male household decision-makers, post-intervention men had significantly higher odds of scoring high on both risk factor (*N* = 1083, OR = 11.72) and symptom knowledge (*N* = 1103, OR = 4.53), compared to their pre-intervention counterparts, after adjusting for country, age, education, and marital status.

Some determinants were associated with cervical cancer awareness and lifetime cervical cancer screening (see supplementary table S2). Women with primary education or higher, showed smaller improvements in risk factor knowledge than those with no education. Similarly, women who reported previous cervical cancer screening showed smaller improvements in risk factor knowledge than those never screened. Among male household decision-makers, rural Ugandan males had higher cervical cancer awareness improvements than rural Bangladeshi males.

### 3.3 Sensitivity analysis

The results between the imputed dataset analysis and the complete case analysis were comparable and consistent. Both found significantly higher odds of scoring high in cervical cancer awareness domains in both females and male household decision-makers (supplementary table S2). A post-hoc interaction model testing the pre/post-intervention×country interaction was conducted for both outcomes in female and male models (table S3). No significant interaction was observed in all models, except for the female risk factor knowledge outcome model (fig. S1). However, post-intervention improvements were observed in both countries, and pooled estimates represent the average intervention effect across settings.

## 4 Discussion

This study evaluated a cervical cancer awareness intervention that combined mass media and CHW-led outreach, tailored to local contexts in two distinct LRS (rural Uganda and rural Bangladesh) among both women and male household decision-makers. We hypothesised that this comprehensive tailored intervention would improve cervical cancer awareness (measured as risk factor and symptom knowledge). Our hypothesis was supported, in that both women and male household decision-makers living in LRS had significantly higher cervical cancer awareness following this intervention.

The results align with a systematic review about interventions in rural areas that focus on improving women's cervical cancer awareness (Zhang et al., 2022). However, only one previous study has assessed cervical cancer awareness change among men in LRS. Vahabi and colleagues (Vahabi et al., 2023) reported improvements in both men's and women's awareness after a CHW-led program in rural India, though women's attitudes toward cervical cancer decreased, possibly due to entrenched gender norms and sociocultural stigma. This can be alleviated through broader community engagement using mass media (Bawuah et al., 2025; Habila et al., 2021). However, this approach was not included in their study, as the intervention took place at a study site. Their results are common challenges to community-based cervical cancer interventions as women often rely on other women for health information in LRS (Habila et al., 2021). This often leads to inaccurate information due to community misconceptions and myths (Habila et al., 2021). Mass media channels such as radio can reach the most remote communities with limited electricity or internet (Bawuah et al., 2025; Habila et al., 2021). In many communities, it is part of the culture for people to gather in the evenings to listen to the radio together (Habila et al., 2021). This might explain why the greatest improvements in our study were found among women with no education, no previous cervical cancer screening, and who had lower pre-intervention cervical cancer awareness.

There are several strengths to our study. We studied a unique intervention that targeted both women and male household decision-makers in LRS through a combination of mass media and CHW-led outreach. Our study is innovative, in that it is one of the few studies that quantitatively evaluates both women's and men's post-intervention cervical cancer awareness. In doing so, we demonstrate improvements in awareness among males, who play a pivotal role in women's cervical cancer screening but generally lack awareness of cervical cancer (Maseko et al., 2024). While male household decision-makers' role are central, in Bangladesh, mothers-in-law also strongly influence women's health decisions. Despite important contextual differences, the consistency of effect across two continentally diverse LRS supports the applicability of this approach across diverse settings. The study could have benefited from measuring the relative exposure of specific intervention components in their contribution to observed awareness improvements. We also demonstrate the feasibility of using the adapted AWACAN to measure awareness in different LRS. This addresses a common gap noted in systematic reviews of the overuse of self-developed unvalidated questionnaires which have shown high heterogeneity and make it difficult to perform meta-analyses (Zhang et al., 2022; Makadzange et al., 2022). Additionally, the study's sample size is larger than those in previous LRS interventions (Zhang et al., 2022; Maseko et al., 2024; Makadzange et al., 2022), which increases the confidence in our findings. However, the results should be interpreted with some caution. Analyses were conducted at the individual level because household-level identifiers were not consistently available for dyadic linkage across settings, limiting insight into intra-household decision-making processes underlying awareness change. Women's health decision-making status was included as a covariate in female models to partially capture decision-making context. The dichotomisation of outcome measures presents inherently less granular information than modelling the full score distribution; however, given the pronounced floor effect and resulting data sparsity, binary logistic regression remained the most suitable analytical choice. It is possible that lifetime cervical cancer screening at post-intervention may have been overestimated, as women may have conflated the lifetime screening question with the PRESCRIP-TEC implementation of hrHPV self-sampling, which was only assessed in this post-intervention group. As the pre- and post-intervention surveys sampled different individuals, observed differences may partly reflect sampling variation rather than the intervention alone; the higher proportion of female respondents at post-intervention may have influenced comparability despite statistical adjustment, potentially overestimating intervention effects and limiting causal inference relative to repeated-measures design. Nevertheless, the quasi-experimental design offers a practical and feasible design to reach hard-to-reach LRS, especially when long-term follow-up is not possible. It allows for timely evaluation of interventions under logistical and time constraints, which limited the number of AWACAN invitations to male household decision-maker respondents at the post-intervention stage. However, sustainability of awareness improvements beyond the study period and behavioural changes (e.g., long-term screening engagement) warrant future investigation.

Pre-intervention cervical cancer screening prevalence was comparable to published estimates for both countries (Bruni et al., 2022). After provision of hrHPV self-sampling, 73.4% of women in the study population participated in cervical cancer screening, meeting the World Health Organization's screening target (World Health Organization, 2021). This aligns with evidence on the effectiveness of hrHPV self-sampling in increasing uptake (Makadzange et al., 2022; John-Akinola et al., 2022).

Around 88% of women reported not being the sole decision-maker of their health. Male involvement is known to be a key facilitating factor to cervical cancer screening uptake (Maseko et al., 2024). These improvements suggest that engaging households and communities through a comprehensive approach can address awareness gaps and increase demand for screening. Combining mass media, already embedded in community life, with CHW outreach offers a feasible and accessible way of disseminating cervical cancer information. This is necessary to supplement the challenges of CHW outreach efforts, such as high turnover which affect trust and long-term sustainability of cervical cancer screening uptake (O'Donovan et al., 2019; Smithwick et al., 2023).

## 5 Conclusions

In conclusion, this study demonstrates that involving male household decision-makers and engaging communities through a culturally tailored, comprehensive intervention using mass media and CHW outreach can effectively improve cervical cancer awareness among both women and male household decision-makers in two diverse LRS. Such an intervention can serve as a model for future implementation efforts and policy to reduce within-country disparities. Prioritising awareness and screening in LRS is a practical strategy for policymakers, as these areas carry a disproportionately high share of disease burden (Garg et al., 2025; Zhang et al., 2022). Exploring long-term effectiveness and cost-effectiveness of such an intervention will inform policy, particularly in LRS.

## CRediT authorship contribution statement

**Fareezah Abdul Karim Siddique:** Writing – review & editing, Writing – original draft, Visualization, Validation, Methodology, Funding acquisition, Formal analysis, Data curation, Conceptualization. **Janine de Zeeuw:** Writing – review & editing, Writing – original draft, Validation, Supervision, Project administration, Methodology, Investigation, Data curation, Conceptualization. **Jaap A.R. Koot:** Writing – review & editing, Writing – original draft, Validation, Supervision, Resources, Methodology, Investigation, Funding acquisition, Data curation, Conceptualization. **Carolyn Nakisige:** Writing – review & editing, Resources, Project administration, Methodology, Investigation, Data curation. **Naheed Nazrul:** Writing – review & editing, Resources, Project administration, Methodology, Investigation, Data curation. **Dan Atukonyera:** Writing – review & editing, Project administration, Methodology, Data curation. **Geertruida H. de Bock:** Writing – review & editing, Writing – original draft, Validation, Supervision, Project administration, Methodology, Conceptualization.

## Declaration of competing interest

The authors declare the following financial interests/personal relationships which may be considered as potential competing interests: The PRESCRIP-TEC project received funding from the European Union’s Horizon 2020 research and innovation program grant agreement No. 964270 and from the Ministry of Science and Technology, Department of Biomedical Technology in India, grant No 13213, under the Global Alliance for Chronic Diseases. The Cepheid company made part of the laboratory equipment available for the PRESCRIP-TEC research in Bangladesh and Uganda, but was not involved in the research design, data collection, or analysis. This study was supported by the Graduate School of Medical Sciences, University Medical Center Groningen, University of Groningen in 2024. Fareezah Abdul Karim Siddique received a personal grant from the University Medical Center Groningen to conduct this research. The funding agencies of the study had no role in study design, data collection, data analysis, data interpretation, or writing of the report. All authors declare that neither they or their institutions have received direct funding from industry for this or any other research project.

## Data Availability

I have shared the link to the data in the attach files step DataversePRESCRIP-TEC – Companion Dataset: Combined Country (Post-Implementation and Follow-Up) (Original data)
