# Supplementary material for: Effectiveness of community-based culturally tailored cervical cancer awareness interventions among women and decision-making men in low-resource settings: A pre-post design evaluation in rural Uganda and Bangladesh
Source: Prev Med Rep. 2026 Jul 15;69:103575. doi: 10.1016/j.pmedr.2026.103575 (PMC13400198; doi:10.1016/j.pmedr.2026.103575)
Supplement: Supplementary file 1 — Supplementary material [file mmc1.docx]

**Supplementary Materials**

**Contents**

| **Details of intervention activities per component** | Page 2 – 3 |
| --- | --- |
|  |  |
| **Supplementary Table 1** | Page 4 |
| Univariate analysis among females and male household decision-makers across low-resource settings in Uganda and Bangladesh (2022-2023) |  |
|  |  |
| **Supplementary Table 2** | Page 5 – 6 |
| Multivariable analysis of factors associated with cervical cancer awareness among females and male household decision-makers across low-resource settings in Uganda and Bangladesh. (Adjusted Odds Ratios, 95% Confidence Intervals) (2022 – 2023) |  |
|  |  |
| **Supplementary Table 3** | Pages 7 – 8 |
| Sensitivity analysis: Multivariable analysis of factors associated with cervical cancer awareness including pre-post intervention*country interaction among females and male household decision-makers across low-resource settings in Uganda and Bangladesh (Adjusted Odds Ratios, 95% Confidence Intervals) (2022 – 2023) |  |
|  |  |
| **Supplementary Figure 1** | Page 9 |
| Simple slopes plot of adjusted predicted probabilities of risk-factor knowledge among women by pre- and post-intervention status and country (pre-post intervention×country interaction model, 2022-2023) |  |
|  |  |

**Intervention Details**

The community mobilisation strategy of PRESCRIP-TEC used the Social Ecological Model of communication. Socio-ecological models were developed to further the understanding of the dynamic interrelations among various personal and environmental factors. The different levels of the layered model include:

- Individual level: affecting an individual’s information, motivation, ability to act, and his or her norms.
- Interpersonal level: triggering individual change through interpersonal communication among and support by partners, peers, family, or a health care provider.
- Community, organization, and service level: impacting community norms, and how members perceive products and services through community groups, networks, associations, and clubs, as well as opinion leaders.
- Enabling environment or societal level: affecting individual and group norms, attitudes and opinions, and laws and policies through media and policymakers.

The following specific communication efforts were conducted for community sensitisation and is organised by mass media and CHW-led outreach components.

**Mass media component:**

- **Weekly talk shows in local radios (Uganda):** In Kakumiro district, Uganda, radio is the predominant media platform. PRESCRIP-TEC secured a 45-minutes slot to address reproductive and sexual health, including discussions on cervical cancer symptoms and prevention. The program featured an entertainer and a health mediator introducing the day's topics, followed by a segment where listeners could express doubts and pose questions, fostering interactive engagement within the community.^[[1]](#footnote-1)^
- **Dedicated landing pages on PRESCRIP-TEC website:** One landing page for both countries was created to gather practical information related to community sensitisation and to serve as a supporting tool for the health ambassadors in the countries^[[2]](#footnote-2)^
- **Posters in concentration areas:** Tailored posters for each country, considering the specificities and cultural context in each case. These posters were prominently displayed in visible locations such as health and community centres by the target communities.
- **Advertising on newspapers:** Paid advertising including a featured news article in the Daily Monitor and the New Vision.^[[3]](#footnote-3)^ Two Ugandan newspapers with wide distribution in Kakumiro district.
- **Miscellaneous:** Social media posts, YouTube short videos, roll-ups, and banners. Flyers and brochures were designed and distributed during the awareness talks and made available to the target audience in health centres and other frequented locations.^[[4]](#footnote-4)^

**CHW-led outreach component:**

- **Community awareness sessions:** PRESCRIP-TEC conducted sensitisation outreaches in the villages of the beneficiary communities to raise awareness about the importance of cervical cancer prevention. These sessions were facilitated by one or several community health workers who had undergone prior training. The number of participants varied, ranging from approximately 10 to 40 people depending on the local context.
- **Theatrical performances (Bangladesh):** In Bangladesh, storytelling was used through theatrical performances to raise awareness among communities. These performances, both highly entertaining and informative, effectively reinforced messages about cervical cancer prevention for the local communities.^[[5]](#footnote-5)^
- **Sessions with opinion leaders (Bangladesh):** Keeping local stakeholders (e.g., religious leaders and influential figures) informed and involved in community mobilisation. These leaders organised neighbourhood meetings with household decision-makers.
- **Door-to-door home visits:** The community health workers also conducted individual home visits to inform beneficiary women and identified household decision-makers (husbands or family members) about the importance of prevention to avoid cervical cancer and the opportunity for the women to perform the high-risk HPV self-sampling.

**Table S1. Univariate analysis among females and male household decision-makers across low-resource settings in Uganda and Bangladesh (2022 – 2023)**

| Females | | *OR* | *[95% CI]* |
| --- | --- | --- | --- |
| Risk Factor Knowledge | | | |
|  | Pre/post (post-intervention) | 6.46 | [5.24, 7.97] |
|  | Country (Uganda) | 1.05 | [0.88, 1.25] |
|  | Age (≥ 36 years) | 0.92 | [0.77, 1.09] |
|  | Education (primary education or higher) | 1.06 | [0.88, 1.27] |
|  | Marital status (Partner) | 1.43 | [0.93, 2.18] |
|  | Health Decisionmaker (Myself) | 0.69 | [0.53, 0.90] |
|  | Lifetime Cervical Cancer Screening (Ever screened) | 0.65 | [0.53, 0.80] |
| Symptom Knowledge | | | |
|  | Pre/post (post-intervention) | 6.37 | [5.22, 7.78] |
|  | Country (Uganda) | 1.37 | [1.15, 1.64] |
|  | Age (≥ 36 years) | 0.82 | [0.68, 0.98] |
|  | Education (primary education or higher) | 1.29 | [1.06, 1.56] |
|  | Marital status (Partner) | 1.56 | [1.03, 2.37] |
|  | Health Decisionmaker (Myself) | 0.84 | [0.64, 1.10] |
|  | Lifetime Cervical Cancer Screening (Ever screened) | 2.03 | [1.61, 2.54] |
|  | | | |
| Male household decision-makers | | *OR* | *[95% CI]* |
| Risk Factor Knowledge | |  |  |
|  | Pre/post (post-intervention) | 12.24 | [8.63, 17.35] |
|  | Country (Uganda) | 1.72 | [1.34, 2.19] |
|  | Age (≥ 40 years) | 1.14 | [0.88, 1.46] |
|  | Education (primary education or higher) | 2.58 | [1.96, 3.39] |
|  | Marital status (Partner) | 2.81 | [1.52, 5.19] |
| Symptom Knowledge | |  |  |
|  | Pre/post (post-intervention) | 4.21 | [3.19, 5.57] |
|  | Country (Uganda) | 1.55 | [1.21, 1.97] |
|  | Age (≥ 40 years) | 1.12 | [0.88, 1.43] |
|  | Education (primary education or higher) | 1.49 | [1.14, 1.97] |
|  | Marital status (Partner) | 1.40 | [0.86, 2.29] |

***Note.*** *OR = odds ratio; CI = confidence interval; 95% confidence interval derived from univariate logistic regression*

**Table S2. Multivariable analysis of factors associated with cervical cancer awareness among females and male household decision-makers across low-resource settings in Uganda and Bangladesh. (Adjusted Odds Ratios, 95% Confidence Intervals) (2022 – 2023)**

|  | | Multivariable Analysis on imputed missing data | | Multivariable complete-case sensitivity analysis | |
| --- | --- | --- | --- | --- | --- |
| Females | | OR | [95% CI] | OR | [95% CI] |
| Risk factor knowledge | | (N = 2140) | | (N = 2026) | |
|  | Pre/post (post-intervention) | 9.51 | [7.52, 12.02] | 10.16 | [7.94, 13.00] |
|  | Country (Uganda) | 1.20 | [0.97, 1.48] | 1.17 | [0.94, 1.45] |
|  | Age (≥ 36 years) | 1.02 | [0.84, 1.24] | 1.01 | [0.83, 1.23] |
|  | Education (primary education or higher) | 0.74 | [0.60, 0.92] | 0.74 | [0.60, 0.91] |
|  | Marital status (Partner) | 0.94 | [0.58, 1.55] | 0.83 | [0.52, 1.33] |
|  | Health Decisionmaker (Myself) | 0.90 | [0.66, 1.23] | 0.83 | [0.60, 1.15] |
|  | Lifetime Cervical Cancer Screening (Ever screened) | 0.32 | [0.25, 0.40] | 0.30 | [0.24, 0.38] |
|  | Constant | 0.29 |  | 0.33 |  |
| Symptom Knowledge | | (N = 2139) | | (N = 2026) | |
|  | Pre/post (post-intervention) | 6.24 | [5.03, 7.74] | 6.37 | [5.10, 7.96] |
|  | Country (Uganda) | 1.20 | [0.97, 1.48] | 1.19 | [0.96, 1.48] |
|  | Age (≥ 36 years) | 0.89 | [0.73, 1.10] | 0.87 | [0.71, 1.07] |
|  | Education (primary education or higher) | 0.84 | [0.68, 1.05] | 0.85 | [0.68, 1.05] |
|  | Marital status (Partner) | 1.16 | [0.73, 1.82] | 1.11 | [0.69, 1.79] |
|  | Health Decisionmaker (Myself) | 1.03 | [0.76, 1.41] | 1.08 | [0.78, 1.50] |
|  | Lifetime Cervical Cancer Screening (Ever screened) | 1.19 | [0.92, 1.53] | 1.21 | [0.94, 1.56] |
|  | Constant | 0.44 |  | 0.46 |  |
|  | | | | | |
| Male household decision-makers | | OR | [95% CI] | OR | [95% CI] |
| Risk factor knowledge | | (N = 1083) | | (N = 1038) | |
|  | Pre/post (post-intervention) | 11.72 | [8.03, 17.10] | 11.89 | [8.13, 17.40] |
|  | Country (Uganda) | 1.91 | [1.40, 2.60] | 1.90 | [1.39, 2.61] |
|  | Age (≥ 40 years) | 0.86 | [0.63, 1.18] | 0.85 | [0.62, 1.15] |
|  | Education (primary education or higher) | 1.14 | [0.81, 1.61] | 1.11 | [0.79, 1.55] |
|  | Marital status (Partner) | 1.88 | [0.97, 3.63] | 1.85 | [1.05, 3.24] |
|  | Constant | 0.26 |  | 0.27 |  |
| Symptom knowledge | | (N = 1103) | | (N = 1055) | |
|  | Pre/post (post-intervention) | 4.53 | [3.31, 6.19] | 4.62 | [3.37, 6.33] |
|  | Country (Uganda) | 1.60 | [1.20, 2.12] | 1.60 | [1.20, 2.13] |
|  | Age (≥ 40 years) | 0.99 | [0.74, 1.31] | 0.98 | [0.75, 1.30] |
|  | Education (primary education or higher) | 0.81 | [0.58, 1.12] | 0.78 | [0.57, 1.06] |
|  | Marital status (Partner) | 1.05 | [0.62, 1.78] | 1.00 | [0.59, 1.69] |
|  | Constant | 0.64 |  | 0.68 |  |

***Note.*** *OR = odds ratio;* *CI = confidence interval; 95% confidence interval derived from multivariable logistic regression.*

**Table S3. Sensitivity analysis: Multivariable analysis of factors associated with cervical cancer awareness including pre-post intervention×country interaction among females and male household decision-makers across low-resource settings in Uganda and Bangladesh (Adjusted Odds Ratios, 95% Confidence Intervals) (2022 – 2023)**

|  | | Multivariable analysis on imputed missing data | | Multivariable complete-case sensitivity analysis | |
| --- | --- | --- | --- | --- | --- |
| Females | | OR | [95% CI] | OR | [95% CI] |
| Risk factor knowledge | | (N = 2140) | | (N = 2026) | |
|  | Pre/post (post-intervention) | 21.15 | [14.11, 31.70] | 21.59 | [14.37, 32.44] |
|  | Country (Uganda) | 3.02 | [1.97, 4.64] | 2.94 | [1.91, 4.54] |
|  | Age (≥ 36 years) | 0.96 | [0.78, 1.17] | 0.95 | [0.77, 1.16] |
|  | Education (primary education or higher) | 0.67 | [0.54, 0.83] | 0.68 | [0.55, 0.84] |
|  | Marital status (Partner) | 1.05 | [0.67, 1.65] | 0.99 | [0.62, 1.56] |
|  | Health Decisionmaker (Myself) | 0.81 | [0.59, 1.10] | 0.78 | [0.57, 1.08] |
|  | Lifetime Cervical Cancer Screening (Ever screened) | 0.35 | [0.28, 0.45] | 0.34 | [0.27, 0.43] |
|  | Pre/post (post-intervention)*Country(Uganda) | 0.28 | [0.17, 0.46] | 0.28 | [0.17, 0.46] |
|  | Constant | 0.16 |  | 0.17 |  |
| Symptom Knowledge | | (N = 2139) | | (N = 2026) | |
|  | Pre/post (post-intervention) | 5.77 | [4.17, 7.98] | 5.71 | [4.12, 7.90] |
|  | Country (Uganda) | 1.11 | [0.78, 1.57] | 1.05 | [0.74, 1.50] |
|  | Age (≥ 36 years) | 0.90 | [0.74, 1.10] | 0.89 | [0.72, 1.09] |
|  | Education (primary education or higher) | 0.85 | [0.68, 1.06] | 0.86 | [0.69, 1.08] |
|  | Marital status (Partner) | 1.15 | [0.72, 1.85] | 1.08 | [0.66, 1.75] |
|  | Health Decisionmaker (Myself) | 1.02 | [0.74, 1.41] | 1.10 | [0.79, 1.54] |
|  | Lifetime Cervical Cancer Screening (Ever screened) | 1.19 | [0.91, 1.55] | 1.18 | [0.91, 1.53] |
|  | Pre/post (post-intervention)*Country(Uganda) | 1.14 | [0.74, 1.78] | 1.23 | [0.79, 1.92] |
|  | Constant | 0.46 |  | 0.50 |  |
|  | | | | | |
| Male household decision-makers | | OR | [95% CI] | OR | [95% CI] |
| Risk factor knowledge | | (N = 1083) | | (N = 1038) | |
|  | Pre/post (post-intervention) | 9.81 | [6.00, 16.04] | 9.86 | [6.03, 16.13] |
|  | Country (Uganda) | 1.72 | [1.21, 2.45] | 1.74 | [1.22, 2.47] |
|  | Age (≥ 40 years) | 0.85 | [0.62, 1.15] | 0.84 | [0.62, 1.14] |
|  | Education (primary education or higher) | 1.11 | [0.79, 1.56] | 1.09 | [0.78, 1.53] |
|  | Marital status (Partner) | 1.73 | [0.98, 3.05] | 1.77 | [1.00, 3.13] |
|  | Pre/post (post-intervention)*Country(Uganda) | 1.52 | [0.73, 3.16] | 1.52 | [0.73, 3.15] |
|  | Constant | 0.30 |  | 0.30 |  |
| Symptom knowledge | | (N = 1103) | | (N = 1055) | |
|  | Pre/post (post-intervention) | 3.69 | [2.40, 5.67] | 3.71 | [2.41, 5.70] |
|  | Country (Uganda) | 1.39 | [0.99, 1.94] | 1.40 | [0.99, 1.96] |
|  | Age (≥ 40 years) | 0.98 | [0.74, 1.29] | 0.98 | [0.74, 1.29] |
|  | Education (primary education or higher) | 0.77 | [0.57, 1.05] | 0.76 | [0.56, 1.04] |
|  | Marital status (Partner) | 0.93 | [0.55, 1.59] | 0.94 | [0.55, 1.60] |
|  | Pre/post (post-intervention)*Country(Uganda) | 1.53 | [0.86, 2.74] | 1.53 | [0.86, 2.73] |
|  | Constant | 0.78 |  | 0.78 |  |

***Note.*** *OR = odds ratio; CI = confidence interval; 95% confidence interval derived from multivariable logistic regression.*

**
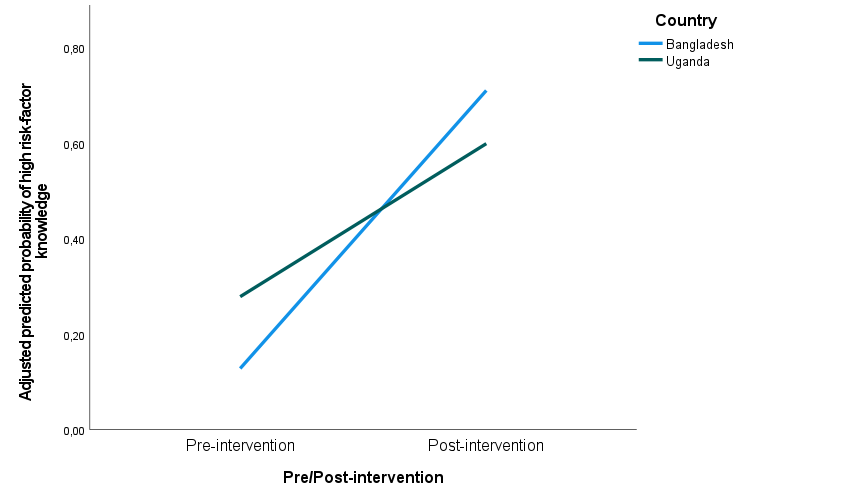
Figure S1. Simple slopes plot of adjusted predicted probabilities of risk-factor knowledge among women by pre- and post-intervention status and country (pre-post intervention×country interaction model, 2022-2023)**

1. <https://x.com/PrescripTec/status/1598542478163992579> [↑](#footnote-ref-1)
2. Uganda: <https://prescriptec.org/community/uganda/>; Bangladesh: <https://prescriptec.org/community/bangladesh/> [↑](#footnote-ref-2)
3. <https://www.newvision.co.ug/category/health/over-1000-women-diagnosed-with-cervical-cance-NV_174218> [↑](#footnote-ref-3)
4. Flyer example in Bangladesh: <https://prescriptec.org/wp-content/uploads/2022/11/flyer-bangladesh.pdf> [↑](#footnote-ref-4)
5. <https://x.com/PrescripTec/status/1584872516178567168/video/1> [↑](#footnote-ref-5)
